# Neonatal intermittent hypoxemia events are associated with later systemic hypertension

**DOI:** 10.1038/s41390-025-03881-w

**Published:** 2025-01-31

**Authors:** Stephanie Martinez, Zhengyi Chen, Juliann M. Di Fiore, Christina Nguyen, Nori M. Minich, Anna Maria Hibbs

**Affiliations:** 1 Division of Neonatology, University Hospitals of Cleveland, Rainbow Babies & Children’s Hospital, Cleveland, OH USA; 2 Division of Neonatology, Department of Pediatrics, College of Medicine, University of Tennessee Health Science Center, Memphis, TN USA; 3 Department of Population and Quantitative Health Sciences, Case Western Reserve University, Cleveland, OH USA; 4 Department of Pediatrics, Case Western Reserve University, Cleveland, OH USA

## Abstract

**Background:**

Approximately 5% of very premature infants delivered at less than 30 weeks’ gestation have systemic hypertension. In adult human and animal models, intermittent hypoxemia events are associated with systemic hypertension. In neonates, intermittent hypoxemia events are associated with adverse outcomes, but it is unknown if they are a risk factor for hypertension. We hypothesize that early intermittent hypoxemia events in very preterm neonates are associated with systemic hypertension at 34–36 weeks’ postmenstrual age.

**Methods:**

Secondary analysis of a single-center cohort study of 164 infants, <31 weeks’ gestational age. Intermittent hypoxemia events were continuously recorded during the first 21 days of age.

**Results:**

There was a significant association between the number of intermittent hypoxemia events (per 100) and systemic hypertension (OR (95% CI) = 1.08 (1.01–1.15)), and both the number of intermittent hypoxemia events (per 100 β (95% CI) = 0.22 (0.10–0.34)) and percent of time with hypoxemia (β (95% CI) = 0.10 (0.01–0.19)) and systolic blood pressure at 34–36 weeks’ postmenstrual age.

**Conclusion:**

This study demonstrated a higher incidence of early intermittent hypoxemia events in preterm infants with hypertension. Decreasing intermittent hypoxemia during this critical period may reduce incidence of later vascular stress in this population.

**Impact:**

- Intermittent hypoxemia events are very common in premature infants and increased frequency of intermittent hypoxemia events is associated with morbidity.
- Intermittent hypoxemia events in adult human as well as adult and neonatal animal models are associated with systemic hypertension.
- This study demonstrated an association between early intermittent hypoxemia events and systemic hypertension in very preterm neonates, adding to the body of literature of possible morbidities caused by intermittent hypoxemia events.
- This study addresses the common, though under-recognized, issue of neonatal hypertension, and suggests increased intermittent hypoxemia events may be contributory.

## Introduction

Systemic hypertension (HTN) of the neonate affects approximately 5% of premature infants delivered at less than 30 weeks’ gestational age (GA) at some point prior to their initial hospital discharge.^1^ Literature suggests a variety of risk factors for HTN in this population, such as the history of placement of an umbilical arterial catheter,^2–6^ acute kidney injury^1,2,4–6^ (AKI), and bronchopulmonary dysplasia^2–8^ (BPD). However, half of premature neonates with HTN have “unexplained HTN” with unclear or possibly multifactorial cause.^6,8^

In the premature neonatal population, there is a high frequency of intermittent hypoxemia (IH) events with an increase in events during the second to fourth week of age, followed by stabilization and gradual decrease.^9,10^ Proposed etiology includes immature respiratory control and movement, concomitant with decreased oxygen stores in the setting of decreased functional residual capacity.^11^ Most IH events peak in the first month of age and decline between 36 and 44 weeks’ postmenstrual age (PMA).^10^ Studies have demonstrated an association between IH events and a range of poor outcomes, including retinopathy of prematurity,^12,13^ BPD,^14,15^ unfavorable respiratory outcome at 40 weeks’ PMA,^16^ prolonged hospitalization,^17^ and cognitive or language impairment at 18 months of age.^13^

The association between IH and HTN has been well-documented in adult humans,^18^ which has been corroborated by numerous murine models implementing chronic IH to elicit HTN.^19–21^ Similarly, a direct link between chronic IH and elevated systemic blood pressure has also been demonstrated in neonatal animal models.^22–26^ It is unknown whether there is an association between IH and neonatal HTN in the preterm human population. In this cohort study, we aimed to determine whether IH events during the first month of age were associated with systemic HTN in preterm infants at 34–36 weeks’ PMA.

## Methods

### Subjects

We conducted a secondary analysis of the Pre-Vent Case Western Reserve University (CWRU) single site study.^27^ Preterm infants <31 weeks’ GA and less than 7 days of age admitted to the UH Rainbow Babies and Children’s Hospital were eligible. Exclusion criteria for the Pre-Vent study included congenital or chromosomal anomalies and those who were unlikely to survive; 175 infants were enrolled at the CWRU site. For this study, infants were included if were enrolled in the Pre-Vent multicenter trial, were living at 36 weeks’ PMA, and had IH data available during the first 3 weeks of age. Parental consent was obtained for the initial prospective Pre-Vent study. For this secondary analysis and additional chart review, the University Hospitals Institutional Review Board approved a waiver of consent. Oversight was provided by the University of Virginia (UVA) Institutional Review Board and an observational and safety monitoring board, appointed by the National Heart, Lung, and Blood Institute (NHLBI).

### Measurements

IH data and key demographic and Neonatal Intensive Care Unit (NICU) morbidities were captured as part of the main Pre-Vent multi-center trial. Per Pre-Vent standardized guidelines,^10^ based on current American Academy of Pediatrics recommended oxygen saturation target,^28^ the oxygen saturation target in the NICU during this study period was 90–95%. Oxygen saturation was continuously recorded (sample rate = 1 Hz, averaging time 8 s) from the clinical bedside pulse oximeter (Masimo, Radical-7, Irvine, CA) using a custom data acquisition system (National Instrument, Hungary and LabVIEW, Austin, TX). IH events were defined as oxygen saturation <90% for 10 s to 5 min using previously validated software (Matlab, Natick, MA)^12^ with IH frequency and total duration <90% assessed during the first 8–21 days of age. Analysis focused on days 8–21 when there is known to be a linear increase in the frequency of IH events.^9,10^

Retrospective chart reviews were performed to capture blood pressure-related data and additional risk factors for HTN. Blood pressures were obtained via unit protocol on one of the four limbs, using an automatic oscillometric blood pressure cuff (Welch Allyn, Skaneateles Falls, NY) or transduced via intra-arterial line, if present. One blood pressure per day was abstracted from a chart review when the infant was between 34 0/7 and 36 0/7 weeks’ PMA. If more than one blood pressure was obtained on a particular day, the blood pressure obtained closest to noon was abstracted. This number of blood pressures was chosen due to the unit norms of obtaining one blood pressure per day in lower acuity patients, which many of these patients were at this PMA. In the event of a mean arterial blood pressure (MAP) not being recorded, the formula [2 × diastolic blood pressure (DBP) + systolic blood pressure (SBP)]/3 was used to calculate the MAP. The median of these recorded blood pressures was calculated and compared to normative values as defined by Dionne et al.^3^ Patients were determined to have “hypertension by norms” if their median SBP or MAP during this period was greater than or equal to the 95^th^ percentile value; that is, if at least 8 of the 15 daily blood pressures were greater than or equal to the 95^th^ percentile.^3^ An infant was deemed to have clinically apparent elevated blood pressures if they met “hypertension by norms” criteria and if clinical progress notes between 34 0/7 to 36 0/7 weeks’ PMA contained one or more of any keywords in relation to blood pressures: “blood”, “elev”, “bp”, “htn”, and “hyperten” to account for misspellings and abbreviations. Data regarding diuretic, anti-hypertensive medication, sedating medication with anti-hypertensive effects, or beta blockers were documented as well.

Information was collected regarding renal ultrasounds obtained prior to 34 0/7 weeks’ PMA. Maximum serum creatinine (SCr) between 14 days of age and 34 0/7 weeks’ PMA was abstracted. Based on previously published studies on surviving premature infants, a normalized SCr cutoff of 0.5 was chosen to define acute kidney injury (AKI).^29^ Patients were identified who had an umbilical arterial catheter (UAC) placed. Study data were collected and managed using Research Electronic Data Capture (REDCap) tools hosted at University Hospitals, supported by the Clinical and Translational Science Collaborative of Northern Ohio, funded by the National Institutes of Health, National Center for Advancing Translational Sciences, Clinical and Translational Science Award Grant, UM1TR004528.^30,31^

### Data analysis

Detectable difference calculation was conducted a priori based on an estimated 160 infants eligible for the study. Setting two-sided significance level at 0.05 and power 80%, we calculated a detectable difference of 0.51 standard deviation in the number of IH events between infants with and without HTN. The detectable difference was estimating 25% prevalence of HTN.

The data characteristics were described by presenting the median (interquartile range) for continuous variables and the frequency (%) for categorical variables, stratified by HTN groups. To account for the non-independence of twins or triplets, we applied generalized estimating equation models (GEE), which can handle correlated data.^32^ We applied GEE logistic regressions for the binary hypertension outcome and GEE linear regressions for the SBP continuous outcome. The univariate differences between HTN groups were assessed using GEE models or Chi-squared tests if GEE model failed to converge. Time series missing value imputation (R package imputeTS) was used in 18 infants to account for missing IH data for the chosen time windows. After imputation, subjects were excluded from the analysis of a particular week if IH variables were missing for more than two days in that week We conducted sensitivity analyses to assess the impact of missing data by analyzing the imputed data and raw data which found similar findings, and therefore imputed data was used. For the patients for whom imputed data was used, it was limited to one to two days only. The percentage difference in effect size for significant results ranged from 1.56% to 17.40%. For both GEE logistic regression model and GEE linear regression, the key predictors were total number of IH events summed over specific time windows (days 8–14 and days 15–21), median of daily percent of time with hypoxemia (SpO_2_ < 90%), and median duration of IH events over specified time windows. Although univariate analysis found GA to have *p*-value 0.21, it was included as a covariate a priori in the model because previous studies have found increased incidence of HTN in lower GA.^1,3^ Other potential confounders were considered for inclusion as covariates in multivariate GEE models: sex, race, small for gestational age (SGA), history of umbilical arterial catheter (UAC), patent ductus arteriosus (PDA) present prior to 34 0/7 weeks’ PMA, and SCr ≥0.5 between 14 days of age and 34 0/7 weeks’ PMA. A covariate was included in the multivariate GEE model if univariate analysis *p* values were <0.10. No covariates were found to meet the criteria of *p* < 0.10, so they were not included in the adjusted models. Analyses were performed using R Language software (Version 4.4.3).

## Results

**Fig. 1:**
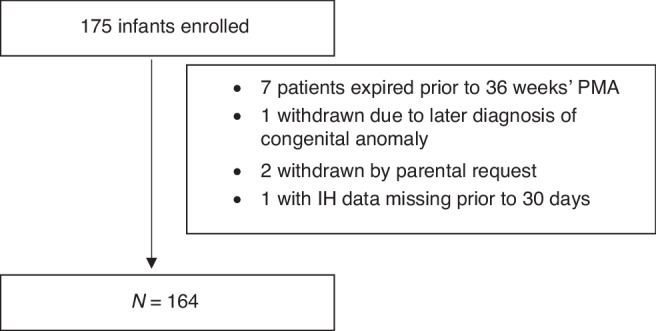
STROBE diagram. One hundred and seventy-five infants were enrolled with 164 infants included in the final data analysis.

**Fig. 2:**
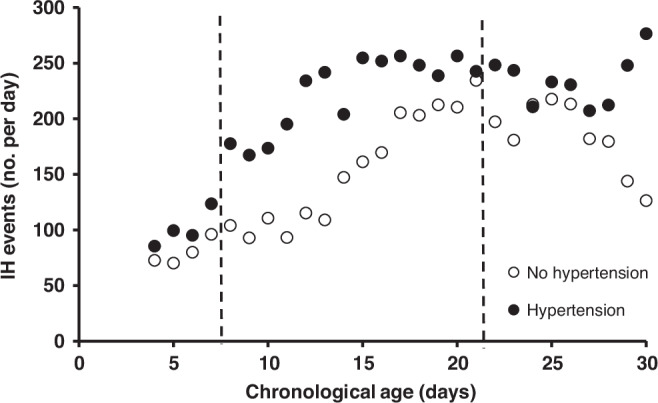
The relationship between the intermittent hypoxemia events (IH) and hypertension. The median number of IH events <90% for infants who did (●) and did not (○) have hypertensive BPs (>95^th^ %ile) at 34–36 weeks’ PMA. Days 7–21, where separation in IH events was greatest, was used in analysis.

**Fig. 3:**
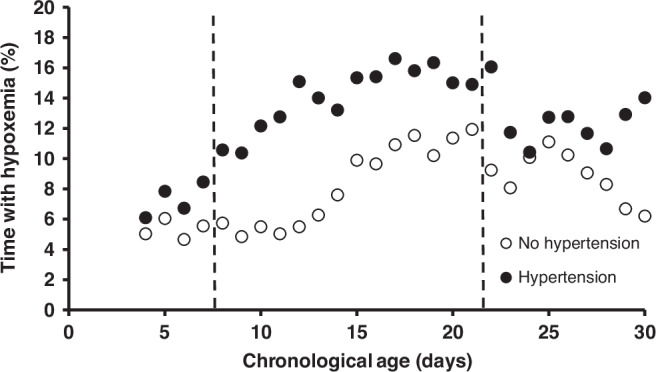
The relationship between overall time with hypoxemia and hypertension. The median percent time with hypoxemia (SPO_2_ < 90%) for infants who did (●) and did not (○) have hypertensive BPs (>95^th^ %ile) at 34–36 weeks’ PMA. Days 7–21, where separation in IH events was greatest, was used in the analysis.

**Fig. 4:**
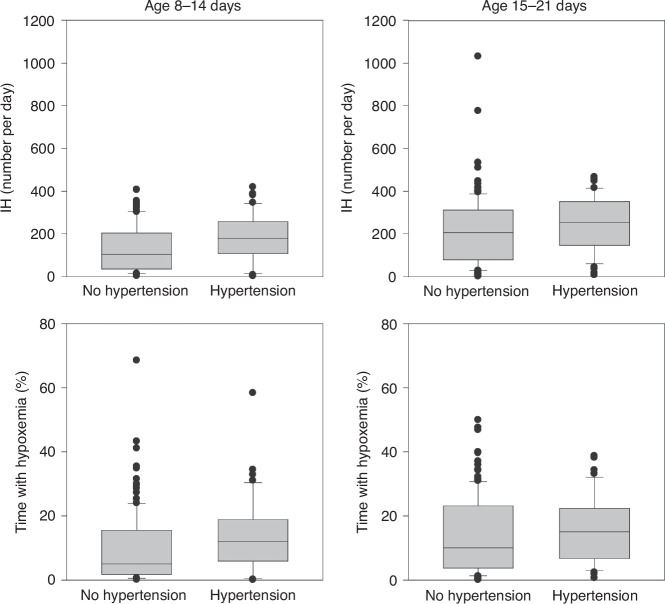
The relationship between intermittent hypoxemia events and overall time with hypoxemia and hypertension with increasing postnatal age. There was a significant (*p* = 0.02) association between the number of IH events 8–14 days of age and later HTN, but no association at days 15–21 (*p* = 0.17). There was no association between the percentage of time with hypoxemia and HTN at either postnatal age.

164 infants met inclusion criteria (Fig. 1). 23.2% (38/164) of enrolled children were found to have HTN by normative ref.^3^ Of these, only 13.2% (5/38) were documented to have clinically apparent elevated blood pressure. There was no difference in antenatal and early hospital course characteristics between those who were and were not found to have HTN by norms at 34–36 weeks’ PMA, including the use of diuretic therapy or other medications with a secondary side effect of lowering blood pressure (such as a beta blocker) (Table 1).When HTN was evaluated against IH parameters stratified by days of age 8–14 and 15–21, there was a significant (*p* = 0.02) association between the number of IH events 8–14 days of age and later HTN, but no association at days 15–21 (*p* = 0.17) (Table 2, Figs. 2 and 4). There was no association between the percentage of time with hypoxemia or the median duration of IH events and HTN (Table 2, Figs. 3 and 4).

**Table 1:** Demographic characteristics and hospital course.

		No HTN (*n* = 126)	HTN (*n* = 38)	*p*-value
**Antenatal characteristics**				
Race (*n* (%))	Black/African American	75/126 (59.5%)	19/38 (50.0%)	0.66*
	White/Caucasian	46/126 (36.5%)	18/38 (47.4%)	
	North American Indian/Native Alaskan	1/126 (0.8%)	0/38 (0.0%)	
	Asian	2/126 (1.6%)	0/38 (0.0%)	
	Mixed	2/126 (1.6%)	1/38 (2.6%)	
Hispanic ethnicity (*n* (%))		5/126 (4.0%)	0/38 (0.0%)	0.48*
Maternal age (median (IQR))		28 (23,33)	28 (23,32)	0.77
Maternal education (*n* (%))	Did not graduate HS	20/126 (15.9%)	2/37 (5.4%)	0.13*
	HS graduate	34/126 (27.0%)	5/37 (13.5%)	
	Any College	35/126 (27.8%)	18/37 (48.6%)	
	Trade School	11/126 (8.7%)	2/37 (5.4%)	
	Associate Degree	11/126 (8.7%)	3/37 (8.1%)	
	College Graduate	11/126 (8.7%)	5/37 (13.5%)	
	Graduate Degree	4/126 (3.2%)	2/37 (5.4%)	
Tobacco during pregnancy (*n* (%))		19/126 (15.1%)	7/38 (18.4%)	0.59
GA weeks (median (IQR))		28.7 (27.0, 29.9)	28.3 (25.6, 29.6)	0.21
GA < 29 weeks (*n* (%))		69/126 (54.8%)	21/38 (55.3%)	0.93
Birth weight (median (IQR))		1120 (838, 1390)	1130 (798, 1405)	0.80
SGA status (*n* (%))		9/126 (7.1%)	2/38 (5.3%)	0.70
Apgar 1 min (median (IQR))		4 (2,6)	4 (2,5)	0.41
Apgar 5 min (median (IQR))		7 (6,8)	7 (5,8)	0.49
Male sex (*n* (%))		62/126 (49.2%)	20/38 (52.6%)	0.67
Multiple birth (*n* (%))		37/126 (29.4%)	7/38 (18.4%)	0.22
Antenatal steroids (*n* (%))		120/125 (96.0%)	34/38 (89.5%)	0.14
Chorioamnionitis (*n* (%))		19/126 (15.1%)	3/38 (7.9%)	0.26
**Hospital course characteristics**				
Received surfactant (*n* (%))		79/126 (62.7%)	28/38 (73.7%)	0.23
UAC present (*n* (%))		53/126 (42.1%)	20/38 (52.6%)	0.24
Sepsis prior to 34 wks’ PMA (*n* (%))		11/126 (8.7%)	3/38 (7.9%)	0.89
Caffeine prior to 34 wks’ PMA (*n* (%))		121/126 (96.0%)	36/38 (94.7%)	0.66
Early HUS IVH Grade 3 or 4 (*n* (%))		6/126 (4.8%)	1/38 (2.6%)	0.59
Chest Tube placed prior to 34 wks’ PMA (*n* (%))		3/126 (2.4%)	1/38 (2.6%)	0.88
PDA diagnosed by echocardiogram prior to 34 wks’ PMA (*n* (%))		36/126 (28.6%)	12/38 (31.6%)	0.66
SCr ≥ 1 between 14 DOL and 34.0 wks’ PMA (*n* (%))		8/126 (6.3%)	2/38 (5.3%)	0.79
SCr ≥ 0.5 between 14 DOL and 34.0 wks’ PMA (*n* (%))		70/126 (55.6%)	21/38 (55.3%)	0.97
Days on oxygen prior to 34.0 wks’ PMA (median (IQR))		19 (6, 41)	31 (6, 51)	0.09
Days with mechanical ventilation prior to 34.0 wks’ PMA (median (IQR))		2 (0,9)	2 (1, 22)	0.37
Renal imaging performed <36.0 wks’ PMA (*n* (%))		22/126 (17.5%)	7/38 (18.4%)	0.89
Renal imaging findings (*n* (%))	Normal	12/22 (54.5%)	6/7 (85.7%)	0.62*
	Echogenicity and pyelectasis	2/22 (9.1%)	0/7 (0.0%)	
	Pyelectasis (no echogenicity)	2/22 (9.1%)	1/7 (14.3%)	
	Echogenicity (no pyelectasis)	4/22 (18.2%)	0/7 (0.0%)	
	Renal Calculi	1/22 (4.5%)	0/7 (0.0%)	
	Hydronephrosis	1/22 (4.5%)	0/7 (0.0%)	
Ventilation at 36 wks’ PMA (*n* (%))	Mechanical ventilation	11/126 (8.7%)	5/38 (13.2%)	0.86
	Non-invasive ventilation	9/126 (7.1%)	2/38 (5.3%)	
	Nasal cannula >2 L	2/126 (1.6%)	2/38 (5.3%)	
	Nasal cannula <=2 L	26/126 (20.6%)	7/38 (18.4%)	
	No respiratory support	78/126 (61.9%)	22/38 (57.9%)	
Oxygen supplementation at 36 wks’ PMA		39/126 (31.0%)	14/38 (36.8%)	0.85
Diuretics at 34–36 wks’ PMA (*n* (%))		28/126 (22.2%)	10/38 (26.3%)	0.50
Clinically apparent BP 34–36 wks’ PMA (*n* (%))		–	5/38 (13.2%)	–

* Chi-squared tests for categorical variables that GEE models did not converge.

**Table 2:** Association between IH at 8–21 days of age and hypertension at 34–36 weeks’ postmenstrual Age: GEE model.

HTN vs No HTN (by norms)						
	No HTN	HTN	Unadjusted		Adjusted^b^	
	Median (IQR)	Median (IQR)	OR (95% CI)	*p*	OR (95% CI)	*p*^b^
# IH events <90%, days 8–14	793.74 (349.37, 1507.65)	1344.89 (961.57, 1932.88)	1.06 (1.02, 1.11)^a^	**0.008**	1.08 (1.01, 1.15)^a^	**0.02**
# IH events < 90%, days 15–21	1475.56 (656.12, 2154.71)	1814.8 (1272.59, 2493.37)	1.03 (1.00, 1.06)^a^	0.09	1.02 (0.99, 1.06)^a^	0.17
# of IH events <90%, days 8–21	2523.28 (1041.31, 3962.89)	3348.4 (2234.62, 4325.37)	1.02 (1.00, 1.04)^a^	**0.03**	1.02 (1.00, 1.05)^a^	0.08
% time with IH <90%, days 8–14	5.04 (1.72, 15.88)	11.96 (6.03, 19.72)	1.03 (1.00, 1.06)	0.08	1.03 (0.98, 1.08)	0.20
% time with IH <90%, days 15–21	9.7 (3.83, 22.76)	15 (6.85, 22.22)	1.02 (0.99, 1.05)	0.27	1.01 (0.97, 1.05)	0.69
% time with IH <90%, days 8–21	7.27 (2.47, 18.83)	13.41 (7.09, 20.47)	1.02 (0.99, 1.06)	0.15	1.02 (0.97, 1.07)	0.43
IH Duration (sec) <90%, days 8–14	29.92 (22.36, 46.21)	35.95 (27.98, 45.99)	1.01 (0.99, 1.04)	0.22	1.01 (0.97, 1.05)	0.63
IH Duration (sec) <90%, days 15–21	27.34 (20.22, 42.57)	31.37 (24.32, 43.82)	1.01 (0.98, 1.03)	0.64	0.99 (0.96, 1.02)	0.60
IH Duration (sec) <90%, days 8–21	28.57 (21.43, 43.38)	33.75 (26.61, 44.7)	1.01 (0.99, 1.03)	0.42	1.00 (0.96, 1.03)	0.85

SBP 34–36 wks. PMA (continuous)						
	<median SBP 34–36 wks. PMA	≥median SBP 34–36 wks. PMA	Unadjusted		Adjusted^b^	
	Median (IQR)	Median (IQR)	Coef. (95% CI)	*p*	Coef. (95% CI)	*p*^b^
# IH events <90%, days 8–14	695.35 (260.8, 1523.84)	1247.45 (733.63, 1669.45)	0.16 (0.06, 0.25)^a^	**0.001**	0.22 (0.10, 0.34)^a^	**0.0004**
# IH events <90%, days 15–21	1380.11 (538.53, 2111)	1664.5 (1144.57, 2387.56)	0.06 (0, 0.13)^a^	**0.048**	0.06 (−0.04, 0.13)^a^	0.08
# of IH events <90%, days 8–21	2250.24 (904.84, 3950.06)	3100.43 (1962.47, 4150.47)	0.05 (0.01, 0.1)^a^	**0.008**	0.06 (0.02, 0.11)^a^	**0.0077**
% time with IH < 90%, days 8–14	3.59 (1.34, 15.74)	9.17 (4.70, 17.88)	0.07 (0.01, 0.14)	**0.03**	0.10 (0.01, 0.19)	**0.03**
% time with IH < 90%, days 15–21	9.49 (3.45, 22.55)	13.02 6.33, 22.2)	0.05 (−0.02, 0.11)	0.17	0.04 (−0.05, 0.13)	0.36
% time with IH < 90%, days 8–21	5.99 (1.95, 18.95)	11.48 (5.85, 20.23)	0.068 (−0.002, 0.138)	0.058	0.09 (−0.01, 0.19)	0.08
IH Duration (sec) <90%, days 8–14	29.89 (21.81, 46.08)	34.43 (24.36, 46.48)	0.04 (−0.01, 0.10)	0.13	0.05 (−0.04, 0.14)	0.27
IH Duration (sec) <90%, days 15–21	27.14 (20.75, 41.8)	31.2 (21.47, 45.24)	0.02 (−0.03, 0.07)	0.49	0.0002 (−0.0780, 0.0783)	1.00
IH Duration (sec) <90%, days 8–21	27.88 (21.41, 43.64)	32.06 (23.38, 43.36)	0.03 (−0.02, 0.08)	0.29	0.02 (−0.07, 0.11)	0.65

Bolded values are significant with *p* < 0.05.

^a^Per 100 events.

^b^Adjusted for GA.

The number of IH events on days 8–14, but not on days 15–21, showed a significant association with the continuous variable of SBP between 34- and 36-weeks’ PMA (days 8–14, *p* = 0.0004; days 15–21, *p* = 0.08). There was a significant association between the percentage of time with hypoxemia on days 8–14 and SBP (*p* = 0.03), but not on days 15–21 (*p* = 0.36). There was no significant association between the median duration of IH events and the continuous variable of SBP (Table 2).

## Discussion

This study found a 23% rate of HTN, higher than previously documented incidence of HTN in neonates.^1–7^ Early IH events between 8 and 14 days of age were associated with HTN and SBP. The relationship between IH events at 15–21 days of age and HTN or SBP did not reach statistical significance. These findings showing a relationship between IH and elevated blood pressure are supported by both animal^24,33^ and adult human^18^ studies. In addition, our findings of statistical significance only with IH events on days 8–14 raise the question as to whether there is a critical window in which IH instigates potential pathways leading to vascular and/or sympathetic changes causing HTN. Missing data could have affected these findings, however sensitivity analysis was performed and had similar results.

Multiple possible mechanisms linking IH and HTN have been proposed, including inflammation, hypoxia-inducible factors (HIFs), reactive oxygen species (ROS), and sympathetic activation.^19,21,25,26^ IH has been shown to cause oxidative stress and generate ROS in both adult animal models of IH,^34–36^ and human infants.^37,38^ IH may also lead to HIF-induced inflammatory vascular remodeling^39^ and endothelial dysfunction.^33,40,41^ A systematic review and meta-analysis of studies of rodent data found exposure to IH increased blood pressures with signs of vascular remodeling including increased intima-media thickness and altered arterial reactivity.^42^ Adult animals exposed to IH have been noted to have increased sympathetic nerve activity,^36,43,44^ and remodeling of end organs innervated by the sympathetic nervous system.^20^ Adult mice exposed to neonatal IH were found to have persistent changes to peripheral baroreceptor^22–24^ and chemoreceptor function mediated by ROS-induced long-term sensory facilitation.^26,45^ An augmented peripheral chemoreflex contributes to sustained activation of the sympathetic nervous system. These data may be significant in the context of the results of the present study since preterm infants with a greater number of apneic episodes exhibited an augmented hypoxic ventilatory response, which is suggestive of enhanced peripheral chemoreceptor activity.^46^ These findings suggest potential multifactorial mechanisms by which IH may potentiate HTN.

The literature suggests multiple risk factors for HTN in premature neonates, including SGA status,^2,6^ PDA,^3,5,7^ UAC,^2–6^ and AKI.^1,2,4–6^ However, none of these were significantly associated with HTN at 34–36 weeks’ PMA in our population. This may be because our study is underpowered to see such a difference. We simplified our definition of AKI to include maximum serum creatinine ≥0.5 based on previously published data related to survivorship from the NICU.^29^ It is possible that a different definition of AKI, such as that used by Kidney Disease: Improving Global Outcome (KDIGO) may have identified alternate infants.^47,48^ Nevertheless, our 50% rate of AKI was similar to that of the Assessment of Worldwide Kidney Injury Epidemiology in Neonates (AWAKEN) study, which used the KDIGO definition.^1^ It has been noted that up to 50% of neonatal HTN are from unknown and multifactorial etiology^6,8^; perhaps IH plays a more impactful role in developing HTN in the preterm neonate than previously recognized.

This study found a higher rate of HTN (23%) compared to the AWAKEN study, which also evaluated raw clinical blood pressure data to define the cohort with HTN (5%).^1^ This large discrepancy could be due to our more frequent sampling (daily vs once per week), though shorter duration (15 days vs entirety of NICU hospitalization), of clinical blood pressure data. Our study used normative values based on Dionne et al.,^3^ while the AWAKEN study used different norms, which may affect the identification of infants with HTN. Of interest, the recently published Recombinant Erythropoietin for Protection of Infant Renal Disease (REPAIReD) study,^49^ which evaluated two-year blood pressure and renal outcomes of infants delivered at 28 weeks’ gestational age, found that 22% of their cohort had elevated systolic blood pressure for age and height. While our study does not follow infants after discharge, the elevated incidence in the study published by Hingorami et al. suggests there could be possible underestimation of hypertension rates in other published literature.

Our study is limited by retrospective chart abstraction of clinically obtained blood pressures, obtained on any of the four limbs, in a variety of infant behavioral states. Interestingly, both the dichotomous variable hypertension/no-hypertension and the continuous variable exploring the association between SBP and HTN were significant, suggesting that even if the 23% identification of HTN in our cohort was over-estimated, there is an association between IH and elevated blood pressures. Furthermore, misclassification (overestimation) of blood pressures due to an agitated state, for example, could bias the estimate towards the null. Nevertheless, our data demonstrated statistically significant association between IH and HTN, despite our data demonstrating a higher-than-expected rate of HTN.

In the current study, only 13% of the values meeting criteria for HTN were documented in daily progress notes (Table 1). This is consistent with the AWAKEN study, where true HTN (based on numeric BP data) was often clinically unrecognized (68% of cases overall, and 84% of cases 30–35 weeks’ GA).^1^ Interestingly, in our cohort, the incidence of infants whose HTN was both clinically recognized and met the criteria of blood pressures ≥95^th^ percentile by normative values at 34–36 weeks’ PMA was 3% (5/164)—similar to other previously published incidence rates of neonatal HTN which did not evaluate raw blood pressure data.^2–7^

Future directions for this research include performing an inclusive prospective study with standardized blood pressure and renal function monitoring. Future studies could also address blood pressures in this population for the duration of and after hospital discharge, even into adulthood. A meta-analysis evaluating prematurity and later outcomes found that low birth weight and prematurity might predispose an individual to metabolic syndrome.^50^ A similar meta-analysis, while not finding increased risk of metabolic syndrome, did find elevated blood pressure in those individuals born preterm.^51^ It would be interesting to further stratify this population by IH events in early life. On a local level, there are opportunities for quality improvement work to recognize and treat hypertensive infants in the NICU.

In summary, this cohort study demonstrated that the number of IH and percentage of time with hypoxemia occurring during the second week of age was associated with systemic HTN at 34–36 weeks’ PMA. The median duration of IH events was not associated with HTN, nor were later IH events (15–21 days of age). These findings suggest there may be a critical window in which IH initiates potential pathologic pathways promoting vascular and/or sympathetic changes leading to HTN. Further studies could be helpful to determine the mechanism behind this association.

## Data Availability

All data generated or analyzed during this study are included in this article. Data is available upon reasonable request with IRB approval. Further inquiries can be directed to the corresponding author.

## Conset for publication

Parental consent was obtained for the initial prospective Pre-Vent study. For this secondary analysis and additional chart review, the University Hospitals Institutional Review Board approved a waiver of consent.
